# Invisible patients in rare diseases: parental experiences with the healthcare and social services for children with rare diseases. A mixed method study

**DOI:** 10.1038/s41598-024-63962-4

**Published:** 2024-06-18

**Authors:** Jan Domaradzki, Dariusz Walkowiak

**Affiliations:** 1 Department of Social Sciences and Humanities, Poznan University of Medical Sciences, Rokietnicka 7, St., 60-806 Poznan, Poland; 2 Department of Organisation and Management in Health Care, Poznan University of Medical Sciences, Poznan, Poland

**Keywords:** Caregiving, Children, Family caregivers, Healthcare services, Parents, Rare diseases, Health policy, Health services, Paediatrics, Public health, Quality of life, Health care

## Abstract

This study explores the experiences of Polish caregivers of children with rare disease (CRD) with health care and social services for CRD. A mixed-methods approach was employed, using an open-ended questionnaire with a convenience sample. Quantitative data presented through descriptive statistics, were complemented by thematic analysis applied to qualitative responses. Responses from 925 caregivers of 1002 children with CRD revealed that the duration of the diagnostic journey varied, spanning from 0 to 18 years, with an average time of 1.7 years. Similarly, the average number of physicians consulted before receiving the correct diagnosis was 4.8. The Internet was basic source of information about children’s disease. Although caregivers were to some extent satisfied with the quality of health care for CRD, they complained at the accessibility of health care and social services, physicians’ ignorance regarding RDs, the lack of co-ordinated care and financial and psychological support. To break the cycle of the diagnostic and therapeutic odyssey that may aggravate the condition of CRD, cause parental stress and financial burden there is a need to change our view on CRD from cure to family-oriented care. Multifaceted challenges and needs of CRD families should be prioritized.

## Introduction

Rare diseases (RDs) are defined in Europe as chronically debilitating or life-threatening conditions with prevalence of less than 50/100000 i.e. 1/2000^1^. Some conditions, however, classified as ultra-rare diseases affect fewer than 1/100000, while hyper-rare diseases affect fewer than 1/100000000^2^. In fact, according to the Orphanet database 84.5% of all RDs have a prevalence of less than 1/1000000^3^. As 250 new diseases are classified as RDs every year, there are currently more than 10,867 RDs^4^, and in the European Union (EU) alone between 17.8 and 30.3 million people live with a RDs, while globally there are between 262.9 and 446.2 million sufferers^1^.

Although over the years clinical breakthroughs have been made in the field of orphan drugs, only between 5 and 10% of RDs have approved drug therapy^5^. RDs are therefore characterised by high morbidity and mortality, especially among children, as 70% of RDs manifest as paediatric diseases, and 50% of children die before the age of five, and 35% die before their first birthday^6^.

Due to the multiplicity, complexity and severity of symptoms resulting in physical, cognitive, mental, behavioural and motoric problems, and the increased risk of premature death, RDs affect the entire family^7–9^, and society as a whole^10,11^. Since RDs have been recognised as a public health priority, they require the integrated care of a range of healthcare professionals^12^. The EU has therefore urged all Member States to adopt a national policy regarding RDs, and has promoted several initiatives, including the improvement of definition, codification and cataloguing of RDs and orphan drugs, the establishment of European Reference Networks for RDs and the development of a European Platform on Rare Disease Registration^13,14^. Some countries are still to adopt national plans for RDs, and others’ policies have technically expired^15^.

Although the number of RD patients in Poland is estimated at between 2.3 and 3 million^16^, the first national *Plan for Rare Diseases* was adopted only recently in August 2021. Its established goal is to improve the visibility of RD patients by creating a Polish Rare Diseases Registry and introducing electronic RD patient passports; to improve the diagnostics and treatment of RDs by facilitating access to modern diagnostics using large-scale genomic testing, medicines, high-quality innovative healthcare services and diets to manage the particular nutritional needs of RDs; and to establish Centres of Expertise for Rare Diseases Information Platform: *Rare Diseases*^16^. Since the plan is still in consultation, many solutions are yet to be implemented. RD families are therefore still waiting for support mechanisms and struggle for appropriate care.

Significantly, most research on RDs in Poland focuses either on its clinical dimension, i.e. the symptoms, causes and treatment options, or RD patients, but less attention has been paid to the family caregivers of children with RDs (CRD). Caring for CRD affects caregivers’ physical, psychological, emotional and functional health, and may undermine the quality of life of both caregiver and CRD^7–9^. Since one of the most onerous challenges parents face in caring for CRD is related to their encounters with the healthcare system^17–26^, this study explores the experiences of Polish caregivers with the healthcare and social services for CRD, by asking the following research questions: 1) What are CRD patents’ experiences with the diagnostic process in their CRD?; 2) How do parents’ perceive health care services for CRD?; 3) What are CRD caregivers’ experiences with the social services?; 4) What are the main needs of CRD caregivers?

## Methods

### Study design

As part of a larger project that focuses on the challenges and needs of parents who care for CRD, this study specifically delves into experiences of Polish caregivers with the healthcare and social services for CRD. Since to date there is no registry of paediatric RD patients in Poland and the exact number of CRD is unknown, the study was designed as a self-administered, anonymous, computer-assisted online survey on the CRD caregivers experiences with healthcare and social services^27^.

### Ethical considerations

This study was performed in line with the principles of the Declaration of Helsinki^28^. The study received ethical and research governance approval from the Poznan University of Medical Sciences’ Bioethics Committee (KB – 833/22, 22 October 2022). Before completing the survey all eligible participants signed the informed consent form to participate which explained the purpose of the study and methods used, voluntary, anonymous and confidential character of the survey, and the possibility to withdraw from the study at any time without any implications.

### Participants and setting

Study participants were recruited via a convenience sampling, with the support of several RD foundations, patients’ associations and organisations via their webpages and Facebook. Participants were accepted if they were parents or family members who provided care for CRD (below 18 years of age), were able to use electronic devices and participate in the online survey.

The study was conducted between October 13, 2022 and May 28, 2023 among 925 voluntary participants.

### Research tool

The data reported here derive from an original questionnaire with both closed-ended and open questions that was designed after a thorough analysis of the literature^17–26^. While it is part of a larger study on the experiences of Polish family caregivers of children with a rare disease, this research focuses on the challenges and needs associated with access to the healthcare and social services for CRD.

Because there is no specific tool for the assessment of CRD caregivers’ experiences with healthcare services an ad hoc questionnaire was constructed. Once it was evaluated by a paediatrician who specialises in RDs, a public health specialist and a medical sociologist, it was pre-tested via an online platform with ten caregivers, and then re-evaluated by additional specialists from the same fields which resulted in rephrasing three questions regarding assessment of various healthcare services for CRD.

The questions selected for this study focused on the caregivers’ experiences with the healthcare system, including assistance from government and social institutions, quality of health care, availability of medical specialists’ care, accessibility of medications, financial assistance and medical information, family support from healthcare workers, doctors’ knowledge on RDs, doctors’ communication skills an emotional support, interaction with genetic and psychological clinic. While most questions were explored using closed-ended questions using a 5-Likert scale ranging from 1 (strong dissatisfaction or disagreement) to 5 (high satisfaction or agreement), caregivers were encouraged to develop these issues by answering additional open question regarding theirs experiences with the health care and social services for CRD (“Could you describe, in your own words, your experiences with the health care and social services for children with rare disease?”). In total, 327 caregivers shared their experiences.

### Data collection

The survey was distributed via webpages and Facebook by sending a letter of invitation from the research coordinator (JD) to a number of RD foundations, patients’ associations and organisations. Upon receiving permission to post the online questionnaire, all eligible caregivers were invited to share their experiences by completing the surrey using electronic devices (e.g. computers, tablets or smartphones). The study invitation included the online consent form and before completing the survey respondents were requested to select an “I agree” or “I do not agree” checkbox. Two follow-up messages were sent in January and March. Completing the survey took between 15 and 20 min.

### Data analysis

Before being transferred to the statistical software JASP (Version 0.18.1), the questionnaire data underwent a thorough check to ensure accuracy, completeness, and consistency.

The quantitative data are presented using descriptive statistics. Additionally, mean and standard deviation (SD) with 95% confidence intervals (CI) for the caregiver’s age, time before the child received the correct diagnosis, and the number of physicians consulted before the child received the correct diagnosis were computed using 5000 bootstrap replicates.

While the classical grounded theory was used for the design of this study^29^, the qualitative data were analysed using a content and thematic analysis^30,31^. The qualitative results were selected by employing an inductive approach, which aims at identifying new analytic categories that are not limited to existing theories or research, and classified as thematic categories. Firstly, all responses were read several times for coding purpose, and categorised during familiarisation. Key words, significant phrases and statements describing caregivers’ experiences with the healthcare system were sought. The initial results were assigned preliminary codes with the relevant supporting quotations selected. While the coding was performed by the principal investigator (JD) who is medical sociologist, trained in qualitative research methods, the second researched (DW) helped in the interoperation of the data. Following grounded theory approach during the entire coding process, the researchers remained open to new potential themes and subthemes emerging during the data analysis. A matrix of the codes was then created according to the themes that emerged from caregivers’ responses. These codes were then organised into domains and descriptive results were outlined. The sampling continued until saturation was achieved and no new themes emerged. When the coding was completed both researchers extended notes and discussed generated categories and emerging theory. Any conflicting data was discussed and resolutions were reached within the team. Once agreement had been reached, the final categories were read and analysed using thematic analysis (Supplementary material Table S1).

## Results

### Characteristics of CRD parents

A total of 925 family caregivers caring for 1002 CRD (see: Supplementary material Table S2) completed the survey, of which 93% were mothers (mean age: 37.4; range: 19–72) (Table 1). 92.1% of caregivers provided care for one CRD and 7.9% for two or more. 67% of caregivers reported a higher level of education and 54.6% reported they were unemployed. 79.8% used no external help in caring for their CRD, and 73.1% received care allowance, i.e. a monthly cash payment granted in Poland to those who renounce paid employment to care permanently for a family member who requires special care (2450 Polish zloty = €565).

**Table 1:** CRD parents’ demographic characteristics.

Characteristics	Caregivers N/N (%)
Caregiver’s role	
Mother	860 (93)
Father	57 (6.2)
Other relative (grandmother, sister, legal guardian)	8 (0.8)
Caregiver’s age (in years)	
Range	19–72
Median	37
IQR (1–3)	33–41
Mean (95%CI)	37.4 (37–37.8)
SD (95%CI)	6.1 (5.7–6.4)
Number of children with RDs	
1	852 (92.1)
2 or more	73 (7.9)
Education	
High school or less	305 (33)
University	620 (67)
Professional activity	
Employed	412 (44.5)
Unemployed	505 (54.6)
Pension	8 (0.9)
Number of hours per week you receive extra-curricular help with CRDS	
0	738 (79.8)
1–6	114 (12.3)
7–10	26 (2.8)
11–15	9 (1)
16–20	14 (1.5)
More than 20	24 (2.6)
Care allowance	
Yes	676 (73.1)
No	249 (26.9)

### CRD parents’ diagnostic experiences

Since only 29.3% of CRD were diagnosed during prenatal testing or newborn screening, many caregivers reported problems with timely diagnosis (Table 2). While the time taken by the diagnostic journey varied, ranging from 0 to 18 years with a median of 1 year and a mean of 1.7 years. Additionally, it provides insights into the number of physicians consulted before receiving the correct diagnosis, with a median of 3 and a mean of 4.8.

**Table 2:** CRD patents’ experiences with the diagnostic odyssey.

	N/N (%)
Method of diagnosis	
Prenatal testing	17 (1.8)
Newborn screening	254 (27.5)
Diagnosis based on signs and symptoms	654 (70.7)
Time spent before CRD received correct diagnosis (in years)	
Range	0–18
Median	1
IQR (1–3)	0–2
Mean (95%CI)	1.7 (1.5–1.8)
SD (95%CI)	2.6 (2.3–2.8)
Number of physicians consulted before CRD received correct diagnosis	
Range	1–50
Median	3
IQR (1–3)	1–5
Mean (95%CI)	4.8 (4.4–5.2)
SD (95%CI)	6.2 (5.3–7)
Main source of information on child’s RD	
Family physician	71 (7.7%)
Medical specialist	505 (54.6%)
Genetic counselling centre	169 (18.3%)
Internet	814 (88%)
Academic literature	373 (40.3%)
Local support group	230 (24.9%)
RD foundations, patients’ associations and organizations	338 (36.5%)
Other (support group on Facebook, acquaintances with CRD)	31 (3.4%)

Although 54.6% of caregivers identified medical specialists as the main sources of information on their children’s RD, only 7.7% identified their general practitioner. For 88% of parents the Internet was the basic source of information about their children’s disease.

### Caregivers’ perspective on the health and social service for CRD

Although most caregivers evaluated five dimensions of the Polish healthcare system for CRD rather positively, the majority expressed dissatisfaction with its organization (Table 3). In fact, while more than half of caregivers were satisfied with the quality of healthcare services for their CRD (58% vs 35.2% dissatisfied), doctors’ communication skills (54% vs 35.1%), interaction with genetic clinics (50.7% vs 26.3%) and doctors’ empathy (50.2% vs 37.5%), still nine dimensions of healthcare services were assessed negatively, and most parents reported disappointing experiences with the Polish healthcare system for CRD. In particular, caregivers complained about the lack of financial help (66% dissatisfied vs % 17.5% satisfied), the lack of support from government and social institutions (65.4% vs 20.8%) and the access to medications for CRD (40.2% vs 36.4%). Caregivers criticised the lack of information regarding their children’s disease from physicians (65.2% vs 29.1%), including practical information on the way to deal with the disease (49.5% vs 38.4), and those provided by the system (56% vs 38.3%). They were also dissatisfied with the availability of specialist care (60.9% vs 32%), support from healthcare workers (51.1% vs 37.5%) and doctors’ empathy (48.6% vs 37.5%). These quantitative data were further supported by textual findings from the qualitative findings described below.

**Table 3:** Parents’ assessment of healthcare services for CRD.

	Very bad N (%)	Rather bad N (%)	I do not know N (%)	Rather good N (%)	Very good N (%)
Assistance for CRD families from government and social institutions	260 (28.1)	345 (37.3)	128 (13.8)	170 (18.4)	22 (2.4)
Quality of health care provided to CRD	78 (8.4)	248 (26.8)	63 (6.8)	433 (46.8)	103 (11.2)
Availability of medical specialists’ care (neurologists, geneticists, psychologists) for CRD	214 (23.2)	349 (37.7)	66 (7.1)	244 (26.4)	52 (5.6)
Accessibility of medications for CRD	156 (16.8)	216 (23.4)	216 (23.4)	300 (32.4)	37 (4)
Availability of financial assistance to pay for CRD rehabilitation	290 (31.4)	320 (34.6)	153 (16.5)	141 (15.2)	21 (2.3)
Availability of medical information on child’s RD	268 (29)	250 (27)	53 (5.7)	285 ()	69 (7.5)
Support for family from CRD healthcare workers	168 (18.2)	304 (32.9)	106 (11.4)	300 (32.4)	47 (5.1)
Doctors’ knowledge concerning RD affecting your child	300 (32.4)	303 (32.8)	53 (5.7)	193 (20.9)	76 (8.2)
Doctors provision of practical information regarding how to manage their children’s RD	197 (21.3)	261 (28.2)	112 (12.1)	267 (28.9)	88 (9.5)
Doctors/neurologists/geneticists-family communication skills	94 (10.2)	230 (24.9)	101 (10.9)	400 (43.2)	100 (10.8)
Emotional support provided to caregivers by doctors	155 (16.7)	295 (31.9)	147 (15.9)	268 (29)	60 (6.5)
Doctors’ ability to establish an empathetic understanding of caregivers	109 (11.8)	238 (25.7)	114 (12.3)	382 (41.3)	82 (8.9)
Interaction with genetic clinic	104 (11.2)	140 (15.1)	212 (22.9)	338 (36.5)	131 (14.2)
Interaction with psychological clinic	99 (10.7)	125 (13.5)	425 (45.9)	220 (23.8)	56 (6.1)

### Parental experiences with the healthcare and social services for children with rare diseases

**Figure 1:**
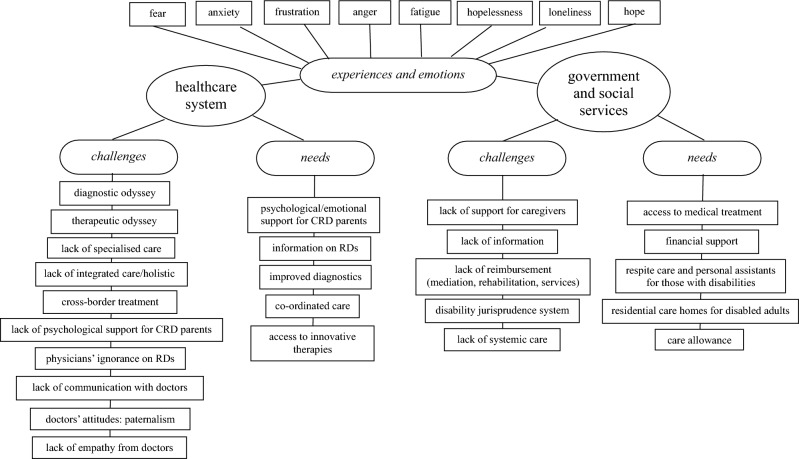
Themes and sub-themes emerging from qualitative analysis.

The thematic analysis of the open question identified two major themes: 1. experiences with the healthcare system, and 2. experiences with the government and social services. Both themes were categorised into two main domains: challenges and needs (Fig. 1).

### Challenges and needs related to navigating the healthcare system

The first theme, with 15 associated sub-themes and representative quotations [Q] is summarised in Table 4. The first six sub-themes related to CRD caregivers’ struggle with the healthcare services.

**Table 4:** Theme 1: CRD caregivers’ experiences with the healthcare system.

Sub-theme	
Challenges	
Diagnostic odyssey	**Q1:** *The most difficult thing is the doctors' lack of interest in my son’s symptoms*—*after the diagnosis, they are all thrown into a syndrome and no one wants to carry out further diagnostics.* (mother, 37) **Q2:** *Currently, doctors refer most sick children with neuro-developmental disorders for an autism diagnosis in order to get them out of the system and not conduct further diagnostics because it is expensive. A child diagnosed with autism has practically no chance of being diagnosed on the National Health Fund (National Health Service), every symptom of the disease is attributed to ASD. I experienced this twice with my children.* (mother, 36) **Q3:** *Doctors prolong everything very much, instead of doing comprehensive WES tests from birth when something is necessary, they are looking in the dark for “What else to come up with?” We are currently waiting for Sotos’ tests, but these are repeated tests because he has already had them done once in this direction, and nothing came of it. We have already had SMA, Prader-Willi and Angelman tests done.* (mother, 28) **Q4:** *We have already wasted too much time in waiting for the diagnosis, passed from physician to physician, where the professor threw up her hands and refused to listen to our doubts (…) We learned the diagnosis by accident, (…) I don’t know what would have happened to this day if it weren’t for the conversation with a stranger who recommended a doctor. There’s a very good chance that we would have visited a few more doctors and our child still wouldn’t be diagnosed.* (mother, 32) **Q5:** *The diagnostic process is one of the worst and most traumatic aspects of the child’s disease, because, as parents, we act intuitively, blindly and often make a lot of mistakes. We don’t have the basic knowledge of where to go or what documents are needed.* (mother, 36)
Therapeutic odyssey	**Q6:** *If you don’t look hard, you won’t find any help for your child. You are alone with everything. No doctor I ever met wanted to be an attending physician.* (mother, 35) **Q7:** *In my area, no one undertook my son’s medical care, so we have to use private medical care (neurologist)*. (mother, 41) **Q8:** *We have been trying to get a diagnosis our son for three years. Doctors don’t listen, they refuse, they take money and don’t refer us any further. Now we have to educate ourselves, look for publications, other parents and children, and demand help or even “specific prescriptions” from doctors. They are not interested in our children, there is no help from anywhere, they do not believe me as a mother when I describe the symptoms. It is a terrible mental and physical burden for the whole family.* (mother, 33)
Lack of specialist care	**Q9:** *Currently, the biggest problems are difficult access to specialists: long queues to specialist doctors and reimbursed rehabilitation.* (father, 38) **Q10:** *Our health service and family doctors are a failure, whatever you can’t do privately, you won’t be able to do at all, or after a year of running around and having to worry about it. For a child psychologist or psychiatrist you have to travel at least 200 km.* (mother, 34) **Q11:** *There are no specialists for rare diseases. Only symptomatic treatment.* (mother, 39)
Lack of integrated care/holistic approach	**Q12:** *There is no holistic approach towards the patients and their caregivers, where many specialists work on one patient and communicate with each other, all having access to the same data/database, including psychologists, physiotherapists and other specialists. In Poland this rarely happens and the caregiver/parent must often repeat information and may omit or forget to whom they gave what.* (mother, 36) **Q13:** *There is no comprehensive approach, we sign up for each specialist separately and it is not entirely clear which specialists we actually need to see.* (father, 41) **Q14:** *Children sometimes have several diseases and parents need to go to several specialists/clinicians.* (mother, 43)
Cross-border health care	**Q15:** *They do not consult each other; they do not send cases abroad.* (mother, 41) **Q16:** *In Poland physicians refused to treat us, they did not perform genetic tests, they provided no instructions, they only said that the child would die anyway after 2 months. There was no empathy, no support, no willingness to help*—*they threw up their hands. In Germany, 10 days after arrival (right away), we already had the results. It was not only the lack of opportunities, because this ties their hands, but above all their giving in and refusing to look further.* (mother, 28) **Q17:** *Virtually nothing is known about our disease. It was first described in 2020. I obtained information from doctors from Germany who described it, so far the knowledge is limited and they are still looking for patients with this gene.* (mother, 38) **Q18:** *My son is not under the care of any doctor in Poland. We get all our knowledge about his disease from a doctor from Italy, whom I found on the Internet based on his publications.* (mother, 30)
Lack of psychological support for CRD parents	**Q19:** *When a parent receives a diagnosis of his or her child’s disease, he or she is left alone to deal with it all. At this point, he or she cannot count on any support from a psychologist because there is no such person in the hospital.* (mother, 42) **Q20:** *Unfortunately, after the diagnosis was made none of the medical personnel offered psychological us, the parents, or our son support.* (mother, 47) **Q21:** *Lack of psychological care and support for parents (…) Actually, no one asked me about it. I received no information about where I could look for support groups. Thanks to the Internet, it may not be difficult to find it yourself, but the fact is that no one mentioned it* (father, 38) **Q22:** *As a family we feel alone, completely alone. There is no support for parents… Psychological support for caregivers of disabled children is very poor, almost non-existent. I have been going privately for 5 years, the visits cost PLN 150 and should be covered by the National Health Fund (National Health Service). Since AA therapy is covered by the National Health Fund, the children's parents should be covered even more.* (mother, 49)
Doctors’ ignorance regarding RDs	**Q23:** *I am terrified by the lack of knowledge among the doctors I have contact with about diseases and such a total lack of understanding of the medical needs of children resulting from their disease*. (mother, 43) **Q24:** *Paediatricians very often have no idea or knowledge about the diseases our children struggle with. It is the parents who explain to the physicians what kind of disease is and how it is treated. Perhaps this is due to the fact that physicians in small towns acquire no new knowledge at all and rely on what they learned ‘centuries’ ago. I once heard a doctor say: “it's your problem that you have a sick child”.* (mother, 42) **Q25:** *Primary care doctors have no idea where to refer patients, like in hospitals and medical staff (midwives, nurses) (…) most people wonder if they can touch her, and what to prescribe, I have to know (…) Many people, including specialists, are afraid of us, we often have no main doctor. The entire responsibility for treatment rests with the parents.* (mother, 33) **Q26:** *If it weren’t our parental instinct and the huge amounts of money spent on private genetic testing, we would never have found out about our son’s rare disease. No doctor even thought to check anything on my son.* (mother, 39)
Lack of communication with physicians	**Q27:** *I also notice that doctors are very formal during conversations. It’s the way they converse, for example it goes like this: “Well, there is no doubt that your son suffers from such a disease, his condition is currently good, you have recommendations in the discharge letter.” I have the impression that I am talking to robots (…) I would expect a calm conversation would be possible, where the doctor would have time to sit down and talk clearly, without going into medical jargon when it is not required.* (father, 38) **Q28:** *Doctors often only answer the questions asked, while I personally don’t always know what to actually ask. Parents receive a lot of information that they cannot process, understand or analyse on their own.* (mother, 38) **Q29:** *Doctors do not provide full consultations regarding treatment and rehabilitation, you have to look for everything yourself* (father, 34)
Doctors’ attitudes: paternalism	**Q30:** *Most often, a visit under the National Health Fund looks like this: doctors do not fully pay attention to parents because they know better, believing that they are doctors and know best.* (mother, 31) **Q31:** *Doctors do not listen to the parents because they are right and they graduated from medical school, not the parent, where unfortunately in our case it is always me as the mother who was right and then a stupid explanation and no one admits to the mistake.* (mother, 35) **Q32:** *Until my daughter received the diagnosis, for 11 years I was treated like a madwoman who had invented everything (…) But for 11 years we were not taken seriously, specialists repeatedly crossed boundaries and violated m child’s comfort, causing trauma and problems with further diagnostics.* (mother, 42) **Q33:** *As a caregiver of a child with PKU, I am often treated as an overprotective person.* (mother, 37)
Lack of empathy in doctors	**Q34:** *Doctors and nurses lack empathy, understanding and basic knowledge about my daughter’s disease. We were kicked out of the doctor’s surgery and refused help in casualty just because my mentally disabled child screamed in fear and annoyed the doctors and nurses* (mother, 38) **Q35:** *We are left alone. We only have a Facebook group. Parents from all over the world. (…) There is no support for parents, I am often ignored or picked on because the doctor does not see the problem and the child’s condition getting worse.* (mother, 42) **Q36:** *Parents are left alone to deal with their children’s disease (…) Nobody is interested in caregivers, none of the doctors, whether we can do it or whether we are able to do… we are very often alone.* (mother, 42)
Needs	
Psychological/emotional support for RD parents	**Q37:** *There should be psychological support for families with sick children. (…) Access to psychological counselling immediately upon diagnosis for parents/caregivers.* (mother, 52) **Q38:** *What we miss most is the support of a psychologist after diagnosis.* (mother, 46) **Q39:** *I believe that after receiving information about the child’s diseases, the family should be provided with immediate psychological help. When a child is sick, the whole family is sick.* (mother, 43)
Information on RDs	**Q40:** *Better access to materials and knowledge.* (mother, 30) **Q41:** *There should be courses for caregivers of disabled children.* (mother, 43) **Q42:** *No information for the family about what help is available to them, how PFRoN* [State Fund for the Rehabilitation of the Disabled] *works, *etc*. We have to “fight” for everything ourselves, find time to learn, read*—*we have more knowledge than doctors and rehabilitation therapists.* (mother, 35)
Improved diagnostics	**Q43:** *With such a small child, making a diagnosis should be a priority.* (mother, 28) **Q44:** *Genetic tests performed such as WES, NGS, microarrays, metabolic tests. Before the parent learns about the disease, unfortunately, he or she has to cover a lot of the costs himself.* (mother, 34) **Q45:** *There are few doctors who can accurately diagnose and treat Dravet syndrome. It is difficult to get a referral to a hospital for diagnostic tests at the beginning of the disease. No refunds for genetic tests.* (mother, 30)
Co-ordinated care	**Q46:** *Co-operation between specialists*—*how to proceed holistically, how to support the development of a child with a rare disease. As parents, we usually look for information on our own, wasting valuable time at the start, because then it is too late.* (mother, 36) **Q47:** *Rare disease centres are not focused on rare disease co-ordination. Here is the quotation “after diagnosis, the child’s co-ordinator is the parent.” As a parent without medical or therapeutic training, this is very burdensome.* (mother, 32) **Q48:** *Due to my profession as a doctor, I have other options for access to specialist colleagues than using the National Health Fund (National Health) and private visits. Often these are friendly courtesy. I am my child’s attending physician, and I co-ordinate everything myself. I know from experience that there are no such co-ordinators for sick people. The lack of someone who thinks about everything that might happen in the team, a rare disease and will control and supervise the patient, *etc*. But will also think about parents and caregivers.* (mother, 40)
Access to innovative therapies	**Q49:** *We are waiting for gene therapy and look to the future with hope.* (mother, 35) **Q50:** *Not blocking access to experimental therapies, e.g. mesenchymal stem cell therapies, we paid for it ourselves and suddenly it is taken away from us without asking our opinion and it is decided that it does not help even though there are positive effects.* (mother, 41)

### Diagnostic odyssey

Caregivers were burdened by the diagnostic process (Q1–Q5). It was framed as a ‘waste of time’ (Q3, Q4) leading to misdiagnosis (Q2, Q3). The diagnostic journey was also described as ‘the most traumatic experiences’ in a child’s disease (Q5), either due to doctors’ lack of interest (Q1, Q2) or intuitive or accidental nature of the diagnostic process (Q3–Q5).

### Therapeutic odyssey

Caregivers struggled to receive appropriate treatment for their CRD (Q6–Q8). While they complained that delays in treatment resulted from doctors’ reluctance to care for CRD (Q6–Q7), parents stressed that it fell to them to find such treatment and cover it from a private funds (Q6–Q8).

### Lack of specialised care

Parents found it very challenging to find a specialist for their CRD, either due to a lack of doctors (Q9, Q11), protracted waiting lists for medical specialists (Q9, Q11) or the necessity to travel great distances (Q10).

### Lack of integrated care/holistic approach

Parents complained at the lack of integrated care (Q12–Q14), poor care pathways and communication between specialists whom they visited separately and had to repeat the information all over again (Q13, Q14).

### Cross-border health care

Since some parents were unable to find treatment in Poland (Q17–Q18), they had to look for healthcare services abroad (Q15–Q18). Caregivers suggested that this was a result both of doctors’ lack of knowledge about their child’s disease (Q15, Q17, Q18), and lack of interest or willingness to provide such care (Q16).

### Lack of psychological support for CRD parents

Caregivers were burdened by the lack of emotional support from healthcare providers (Q19–Q22). Some complained at the scant support at the time of diagnosis (Q19, Q20). Others suggested that they had never received any support (Q19, Q21, Q22). Many parents therefore felt abandoned and neglected (Q19, Q22).

Another four sub-themes referred to the difficulties experienced in interacting with doctors.

### Physicians’ ignorance of RDs

One of the greatest challenges parents reported facing was related to healthcare professionals’ lack of knowledge regarding RDs (Q23–Q26). In the face of doctors’ ignorance parents had to become experts in their child’s disease (Q23–Q26). Caregivers reported at the same time that doctors’ ignorance resulted in a lack of understanding of the needs of parents (Q23, Q24) and empathy (Q24, Q25).

### Lack of communication with doctors

Parents complained at doctors’ communication skills and poor interaction (Q27–Q29). They criticised doctors for speaking in an overly formal manner, like ‘robots’ (Q27), devoting too little time to caregivers, and failing to engage in meaningful communication (Q27, Q28). Caregivers also expressed dissatisfaction with the amount of information received and suggested that they had to seek it out themselves (Q28, Q29).

### Doctors’ attitudes: paternalism

Caregivers reported paternalistic attitudes among doctors (Q30–Q33) and suggested that doctors acted as though they knew everything better than parents did and ignored caregivers’ ‘gut instincts’ and suggestions (Q30, Q31). Some parents reported being treated as though they were ‘mad’ or ‘overprotective parents’ (Q30–Q33).

### Lack of empathy from doctors

Parents complained at healthcare professionals’ lack of empathy and compassion (Q30–Q33), and suggested that they cared neither for CRD (Q30, Q31) nor caregivers’ problems and needs (Q32). Parents also reported that such coldness resulted in mistreatment (Q30, Q31).

Another five sub-themes referred to caregivers’ needs related to healthcare services for CRD.

### Psychological/emotional support for CRD parents

Parents expressed the need for psychological support (Q37–Q39). While stressing that a child’s disease affects the entire family (Q39), caregivers emphasised that emotional and psychological support is required as early as at the time of diagnosis (Q37–Q39).

### Information on RDs

Parents reported the need for information on the RD their child had been diagnosed with (Q40–Q42). They expected ‘materials and knowledge’ on the disease itself, (Q40, Q42) and practical information about where they might seek institutional help (Q42). Courses teaching parents how to care for their RD child were also requested (Q42).

### Improved diagnostics

Another need referred to early diagnosis, which would help parents cope better with the stress (Q43–Q45). While prompt diagnosis was defined as ‘a priority’ (Q43), parents expressed the need for better access to modern diagnostics using large-scale genomic testing (Q44), and stressed the prohibitive expense of covering it from private funds (Q44, Q45).

### Co-ordinated care

Caregivers hoped for improved co-ordination of care for their CRD (Q46–Q48). In discussing the psychosocial consequences of fragmented care (Q46, Q47) they stressed the urgent need for improved co-operation between specialists (Q46–Q48). The need for an holistic approach towards CRD and their caregivers was also emphasised (Q48).

### Access to innovative therapies

Caregivers were also hoping for a cure for their children (Q49–Q50). While some parents expressed hope for effective gene therapy (Q49), others described their struggle to secure experimental therapies (Q50).

### Caregivers perception of the government and social services for CRD

The second theme referring to parents’ experiences with the government and social services, with 11 associated sub-themes and representative quotations, is summarised in Table 5.

**Table 5:** Theme 2: CRD caregivers’ experiences with the government and social services.

Subtheme	
Challenges	
Lack of support for caregivers	**Q1:** *We are invisible to the system. (…) Lack of support in institutions, no psychologist for the family, no place for siblings of sick children where they could feel relief from everyday life.* (mother, 38) **Q2:** *The parent is left alone, no one cares what the child needs.* (mother, 36) **Q3:** *No one cares that* [the child] *screams in pain, a certificate of disability, the pro-life act*—*it has no real impact on getting to specialists. A child may suffer for months.* (mother, 36)
Lack of information	**Q4:** *We don’t have basic knowledge of where to go or what documents are needed. Everything that to some extent (formally) helps us on our way, such as applying for a disability jurisprudence, early development support, the pro-life law, we learned accidentally thanks to the kindness of people we met along the way, and not thanks to, for example, any institution we went to where someone explained in a factual and patient way what we should do*—*that’s what we really missed.* (mother, 36) **Q5:** *I learned a lot from parents of disabled children, where to go, what kind of re-imbursement, rehabilitation, *etc*. we might apply for. None of the doctors or employees from the MOPS* [Municipal Centre for Family Assistance] or PSPR [District Family Assistance Centre] *gave any directions or informed me what kind of help to seek and where I should go for help with everything.* (mother, 41) **Q6:** *There is no information for the family about what help they can get. Conflicting information about rehabilitation equipment. Failure to inform parents of disabled children about support programmes.* (mother, 27)
Lack of re-imbursement (medication, rehabilitation, services)	**Q7:** *Will there ever be a refund for specialist PKU food? It would be much easier for ill people, a great help because finances are getting worse these days and food is becoming more and more expensive.* (mother, 33) **Q8:** *No refund, sometimes you have to wait 2 years for the National Health Fund and up to 3 months privately*—*how is this possible?* (mother, 34) **Q9:** *Further genetic tests, covering subsequent stages, should be reimbursed, as the costs are high.* (mother, 50) **Q10:** *Rehabilitation equipment is expensive. To get anything, you have to run about a great deal to MOPS* [Municipal Centre for Family Assistance] or *PFRoN* [State Fund for the Rehabilitation of the Disabled]. *There is little rehabilitation. We pay for everything with our own money. The equipment is usually expensive and caregivers cannot afford to buy it. PFRON is a place where people should help the sick, but sometimes they defend themselves with all their might.* (mother, 50)
Disability jurisprudence system	**Q11:** *An important problem is the way case law works in Poland, which is in a deplorable state… even though the child requires my constant care, I fought for 3.5 years to obtain point 7* [disability pension which indicates the need to provide constant care for a child] (mother, 33) **Q12:** *Evaluating physicians and court experts have no idea about rare diseases, and based on their statements and opinions, decisions are made that are harmful to sick children and their caregivers.* (mother, 39) **Q13:** *Disability case law is a joke. The state does nothing to help the sick. Everything that is needed that might help the patient must be paid for from your own funds.* (mother, 29) **Q14:** *The physician adjudicating on the disability commission does not read the medical documentation and considers Kabuki syndrome not to be a progressive disease.* (mother, 41)
Lack of systemic care	**Q15:** *Our state has no decent system that would really support us as parents in the hardships of everyday life. (…) National Health Fund medical care is scandalous, there is no systemic care for DMD patients. The system is not unsupportive as much as it is destructive.* (mother, 39) **Q16:** *There is no complementary and comprehensive support system for families with children with rare disease in Poland. The family is completely neglected, there is no comprehensive guidance and support by a psychologist, psychiatrist and dietician through constant care of the person from the beginning of the disease throughout life. The families have serious emotional problems and have no specialist support in this area.* (mother, 41) **Q17:** *The issue of psychological assistance for caregivers of sick/disabled people is ignored, as is the systemic issue of family support*—*in practice it does not exist. Medical help too*—*you often have to choose between your own health and that of your child, which in turn puts a double burden on us*—*because who will take care of your child when, for example, you have to go to the hospital. We give up on ourselves because we have no “rational” choice.* (mother, 36) **Q18:** *After two years of living in Poland with a disabled child, we decided to find a place where our son has a chance for a better life. We have been living in Norway for 6 years. There is a gap between the approach of Polish and Norwegian doctors. What matters here is not only my son, but also my opinion as a mother. All necessary equipment is fully refunded! Conditions in the hospital are much better. Norway is a caring country that cares about people with disabilities. Poland is far behind. Here you are told to parent your children, and then the parent has to worry about it himself. It’s sad that this is the case.* (mother, 35)
Needs	
Access to medical treatment	**Q19:** *Children with a rare disease have no real priority in their access to medical care, even with a disability certificate and the Pro-Life Act.* (mother, 31) **Q20:** *The waiting time for a medicinal product sometimes exceeds the prescription expiry date.* (mother, 36) **Q21:** *It should be possible to treat children with rare diseases abroad, paid for by the National Health Fund (…) re-imbursement of medicines but also special dietary products (…) food products consume a great deal of money, sometimes impairing the daily needs of healthy people and other family needs that have to be forsaken in favour of a sick child.* (mother, 43)
Financial support	**Q22:** *Why don’t we have free medicines, doctor’s appointments and rehabilitation.* (mother, 49) **Q23:** *Reimbursement for home equipment for monitoring Phe levels and better access to and re-imbursement of specialised PKU food.* (mother, 33) **Q24:** *A lot would be changed by refunds for low-protein food and greater financial assistance from the state, best regards.* (mother, 44) **Q25:** *Psychological support for caregivers of disabled children is very poor, almost non-existent. I have been going privately for 5 years, visits cost PLN 150 and should be covered by the National Health Fund. Since AA therapy is covered by the National Health Fund, children’s parents should be covered even more*
Respite care and personal assistants for those with disabilities	**Q26:** *Respite care*—*a problem with money so that every parent can benefit.* (mother, 38) **Q27:** *I would benefit from respite care in a difficult situation.* (mother, 26) **Q28:** *ON assistant* [personal assistant for those with disabilities] *allows caregivers to help take care of a disabled child. There are no regulations regarding personal assistants for persons with disabilities.* (mother, 45)
Residential care homes for disabled adults	**Q29:** *I am afraid of what will happen to my son when he reaches adulthood and turns 18. There is no centre in Poland for adults with rare diseases. So what’s next? Because the child will not recover miraculously.* (mother, 44) **Q30:** *I have serious concerns about my child’s quality of life after graduation. For profoundly disabled adults with rare diseases, there are very few options available when it comes to rehabilitation and socialisation. They are confined to their homes and have few opportunities for activities and care.* (mother, 32)
Care allowance	**Q31:** *Care allowance should be treated as support in raising a child with a disability certificate and not as the caregiver’s earnings. We should have the opportunity to work legally 3/4 or 1/2 time to earn extra money for the household budget and go out to meet people without the risk of losing benefits.* (mother, 28) **Q32:** *All parents should receive care benefits. Some parents do not receive it because the case law does not award these points. I had to fight in court. This shouldn’t be the case in Poland* (mother, 42) **Q33:** *A caregiver who receives care benefits should be able to work without losing the benefit. The ban on earning extra money deprives us of the right to a decent life.* (mother, 36) **Q34:** *After 16 years of caring for a child, I started working. I am very satisfied with my work. It’s my therapy, my escape from everyday life. Work is a very important element in my life.* (mother, 41)

### Lack of support for caregivers

In describing the health policy towards RDs parents stressed that they felt abandoned (Q1–Q3). They complained at the lack of institutional support (Q1, Q2) and that no one cared about needs of either CRD (Q2, Q3) or parents (Q1, Q2).

### Lack of information

Caregivers felt abandoned by the government and social institutions and stressed the extent to which they felt ill-supported in securing information on medical and social services for their children, including rehabilitation, re-imbursement and system of jurisprudence (Q4–Q6). They emphasised that, when seeking practical easy-to-understand information, parents had to rely more on other parents than social institutions (Q4, Q5).

### Lack of re-imbursement (medication, rehabilitation, services)

Parents complained that the lack of re-imbursement meant that caregiving imposed a substantial economic burden on caregivers and reduced treatment available (Q7–Q10). They described long waiting lists (Q8), high costs of personal expenditure, including genetic testing (Q9), special foods (Q7) and rehabilitation equipment (Q10).

### The disability jurisprudence system

Parents reported problems with the Polish system of disability jurisprudence (Q11–Q14) which was described as being in ‘a deplorable state’ (Q11) and ‘a mockery’ (Q13). They complained at the protracted procedures required to claim disability benefits (Q11) and described ignorance of doctors in the pension commission, who had no knowledge of RDs (Q12, Q14).

### Lack of systemic care

Caregivers emphasised that there was no systemic care for RD families (Q15–Q18). They complained at fragmented care and lack of co-ordination between healthcare providers and services (Q15–Q17). While parents criticised the accessibility of health care and social services (Q15–Q18), they descried the system as ‘unsupportive’ and ‘destructive’ (Q15). Some parents therefore left the country (Q18).

Finally, caregivers reported six main unmet needs.

### Access to medical treatment

Parents hoped for enhanced accessibility to modern RD treatments (Q19–Q21). In stressing that provision of medical care to CRD is often neglected (Q19) they expressed the wish it would take less time to receive treatment (Q20). They also believed that National Health Fund should cover all costs, including treatment abroad (Q21).

### Financial support

Caregivers described difficulties in procurement of medications and healthcare services (Q19–21) and they expressed the need for financial assistance and hoped for re-imbursement for medications, appointments and rehabilitation (Q22). Among other needs they mentioned equipment for rehabilitation, specialised food items (Q22, Q24) and psychological support (Q25).

### Respite care and personal assistants for those with disabilities

Parents expressed the wish for much-needed respite care that would enable them to rest and take time for themselves (Q26–Q28). Money was the problem for some caregivers (Q26) and they also wished for personal assistants for those with disabilities, who would support them in the performance of basic daily activities and decisions regarding care (Q28).

### Residential care homes for disabled adults

Some caregivers anticipated challenges relating to transitioning from paediatric to adult care and one of the most important supportive care needs was the organisation of residential care homes (Q29–30). Parents hoped that such facilities would provide health care for their growing children (Q29, Q30) and company in order to enable them to live life to the best of their potential (Q30).

### Care allowance

Since until recently the right to care benefits in Poland depended on the caregivers’ giving up paid work, parents suggested that it should be legal to receive a care allowance and at the same time take up employment without restrictions (Q31–Q34). They also believed that parents who wish to work should not be deprived of the benefit (Q31, Q32). Caregivers also stressed that benefits stemming from professional activity far outstrip financial income and guarantees better care for their children (Q34, Q35) and emphasised the psychological aspects of work as a coping resource (Q33, Q34).

## Discussion

While several previous studies on the experiences of caregivers, namely parents, of CRDs have been conducted in Poland, including Huntington disease^32–34^ or Prader-Willi syndrome^35^, most often they focuses either on the challenges and needs related to caring for such a child, caregivers’ burden or social functioning of a family whose member experiences RD. However, few data are available on the experiences of CRD caregivers with the healthcare system in Poland^20^. This study is therefore, one of the few Polish studies that highlights the family caregivers’ interactions with healthcare and social service for CRD. This is of key importance because, while Poland adopted its first *Plan for Rare Diseases* only recently, it is yet to be implemented this study shows that many parents struggle with the diagnostic and therapeutic odyssey their children face, and feel neglected by the system.

Thus, even though this study shows that some dimensions of healthcare services for CRD were assessed positively, most caregivers reported negative experiences with Polish healthcare system and were dissatisfied with the way it is organized. However, also previous studies demonstrated that although Polish parents of persons with Huntington disease reported many challenges and negative feelings resulting from caregiving, their perception of burden was mostly influenced by negative experiences with the healthcare system, as 9.1% declared having had positive experiences, while almost 42% described their encounters negaively^20,32^. What is equally important, is that caregivers enrolled in our study also reported difficulties in accessing professional care, complained over lack of resources required for providing care, and expressed the need for support system for CRD families.

While CRD visit doctors more frequently and wait much longer for diagnoses than others^17–23,25,26^, they are also several times more likely to be hospitalised, and the average length of their hospitalisation is longer^24,25^. During hospital stays they also face more difficulties and are more dependent on co-ordinated care involving large, multi-disciplinary teams. Finally, CRD parents report greater reliance on health care, as their children often use a wide variety of primary, secondary and tertiary health services^19,20,24,25^.

This research confirms that medical dealings for many CRD parents are very confusing, chaotic and expensive, and often become a protracted diagnostic odyssey^17–23,25,26^. Libura et al.^36^ showed, however, that more than 60% of those with RDs in Poland have received at least one misdiagnosis, averaging 3.5 misdiagnoses, and almost one quarter have waited ten or more years for their diagnoses.

Simultaneously, although caregivers enrolled in this survey reported that one of the reasons for this is the lack of access to modern diagnostic techniques that use large-scale genomic testing, they also suggested that such delays are caused by a lack of knowledge about RDs among doctors and other healthcare workers^8,17–23,26,37^. This is in line with previous research showing that Polish doctors and nurses have insufficient knowledge of RDs and feels unprepared to deal with patients with RDs^37,38^. In fact, parents often reported knowing more about their children’s disease than doctors and they were burdened by the necessity to take the role of “expert caregiver”, especially since it was frequently unacknowledged by the patronising healthcare professionals who treated them with little emapthy^8,21,25,26^. This study also confirms that, when faced with information deficits, poor communication skills and lack of empathy from doctors, most parents sought information from web-based sources^22,23,25^.

While many caregivers also reported that their children received misdiagnoses or late diagnoses, they also had to consult a number of specialists before the final diagnosis was made^20–22^. This is important because research has demonstrated that the diagnostic odyssey is a great emotional and physical stress for the entire family and may impede adaptation to the disease and the quality of life^7–9^. It also results in unnecessary medication, hospitalisations and uncomfortable and painful treatment, including surgeries that may aggravate the condition of CRD and cause a financial burden^17–26^. Teutsch et al.^24^, for example, demonstrated that, while only 51% of Australian RD families had private health insurance, they spent on average AU$31,000 (US$21,500) annually per child, mainly, on travel costs, medical appointments, medications and medical and assistive equipment. A recent study conducted in France, Germany, Italy, Spain, the UK and the US also estimated the annual direct medical costs in the case of Huntington’s disease at €12,663, indirect medical costs at €2984 and indirect costs at €47,576^11^. Another study estimated the direct healthcare costs of Dravet syndrome per three months as €6043 ± €5825 per patient. They also suggest that total indirect costs were €4399 ± €4989 in mothers and €391 ± €352 in fathers^10^.

This study also supports previous research showing that a diagnosis was not always followed by appropriate care^8,17,18,20,25,26^. While some parents reported being denied treatment for their CRD, most caregivers were ill-informed, either by the specialists or social institutions, about psychosocial care and support available to RD families. While most parents were unaware of psychosocial care services, many reported that there being no such offers available^17,20,25^. Many caregivers reported that doctors focus mainly on medical care but neglect children’s and caregivers’ emotional and psychosocial needs. Although parents expected practical information on the management of the symptoms and treatment, and socio-legal and organisational issues related to the care allowance and re-imbursement system, they were rarely informed about such offers and did not know whom to turn to for such help. They felt alone in their struggle with the system^26^ and turned to online-support groups^23,25^.

Finally, this study confirms that one of the biggest challenges experienced by Polish CRD parents is related to navigating the healthcare and social system^8,17–26,32^. Caregivers reported problems in timely diagnoses and access to specialists, as well as their struggle with a lack of co-ordinated care for CRD, and were burdened by the necessity to take on role of co-ordinator of their children’s care^8^. Since the governmental list of chronic diseases contains only a few RDs, many parents reported problems with socio-legal and organisational care, i.e. access to government support systems and social services. They specified the many challenges to funding for medications, therapy and rehabilitation, which had to be covered from personal funds, denting family finances^8,10,19,25^. CRD carers therefore felt neglected by the system and described their caregiving as a ‘lonely journey’^8,20,23,25,26^.

This, research also shows that according to CRD parents Polish healthcare system often focuses on children and clinical dimension of disease, but often neglects caregivers emotional and psychological needs. While most caregivers enrolled in this study complained over lack of psychological support for CRD families, they also emphasized the need for holistic approach towards RD families and stressed that emotional and psychological support should be available to them from the time of diagnosis through entire disease trajectory and should include entire family.

## Strength and limitations

The unquestionable strengths of this study are that, since there is scarcity of studies describing the experiences of Polish caregivers of CRD with the healthcare and social services, it helps in identifying actions required from the government, medical and social institutions to improve healthcare for CRD families. Another strength is that it included a large sample of caregivers of children with a variety of (ultra)rare diseases. Since many caregivers acknowledged that this survey gave them the opportunity to be heard for the very first time it might also have a therapeutic value.

This study had, however, some limitations Firstly, while 925 caregivers of 1002 CRD responded, the estimated number of CRD cases in Poland is much higher, so the results cannot be extrapolated to the entire population of caregivers of CRD in the country. Nevertheless, it should be acknowledged that although the first *National Plan for Rare Diseases* was adopted in Poland in 2021, there is still no registry of persons with RD, and the exact number of paediatric RD patients is unknown. Furthermore, none of the RD foundations, patients’ associations, and organizations involved in the study were able to provide the number of paediatric RD patients with a given condition in Poland. Secondly, since this study was designed as an online survey, there is a risk that it failed to reach caregivers in remote locations with no access to the internet or those who felt uncomfortable using social media platforms and electronic devices. Thirdly, not all caregivers are members of (online) support groups, associations or organisations that helped in the recruitment process. Fourthly, because this survey focussed on experiences of caregivers of children with RDs, the results may be unrepresentative of the experiences of caregivers providing care over adult persons with RD who often face different challenges and express different needs. Fifthly, even though the questionnaire used in this survey was consulted with three external experts in paediatrics, public health and medical sociology and was pre-tested in a pilot study with ten caregivers, it was not validated. A further limitation includes method of qualitative data collection since only one open question was asked without triangulating it against other sources, i.e. caregivers’ interviews or Facebook posts. Moreover, since only 327 participants provided qualitative data there is a potential participation bias related to the possibility that only dissatisfied caregivers shared their experiences by answering additional open question regarding caregivers’ experiences with the health care and social services for CRD. In fact, even though many participants were somehow satisfied with various dimensions of healthcare services for their CRD, of all responses provided only nine were positive, but all of them referred to parents’ perspective on the impact of their child’s disease on caregivers’ personal growth or emotional relationship with CRD, and not to respondents’ experiences with healthcare system. Since only 57 fathers completed the survey there is also a possible gender bias. Lastly, even though qualitative were translated with the help of bilingual translator, still there is a risk of misinterpretation of language nuances inherent in participants’ experiences and that situational context may be lost.

## Conclusions

In summary, this study highlights that the diagnostic and therapeutic journey that befalls their children means that CRD parents face many challenges with the healthcare system and express numerous unmet needs. As it shows that there is a urgent need to change our perspective on RDs from cure to family-oriented care, some important policy implications therefore emerged from this study. Although they have been also recommended globally, they are particularly relevant in countries like Poland, where health and social policy for RDs is in progress and national *Plan for Rare Diseases* is still to be implemented:

1. There is an urgent need for further exploration into challenges and unmet needs described by caregivers that hinder access to health care and social services for their CRD.
2. In particular, in order to assure timely and accurate diagnoses it is crucial to improve and facilitate access to modern diagnostics using large-scale genomic testing.
3. Since health care for CRD is fragmented and lacks co-ordination between medical specialists, healthcare providers and services, there is a need for integrated care for entire RD families that would consist of multidisciplinary team of specialists, including geneticists, neurologists, psychiatrists, dieticians, clinical psychologists, nurses, physiotherapists and speech therapists.
4. To facilitate the management and organisation of care for CRD rare disease co-ordinators, similar to cancer care co-ordinators that were introduced in Poland in 2015, should be organised, so that they would inform, educate and guide RD families through the healthcare system and help to organise an RD person’s care.
5. Although several Polish research and health institutes and clinical hospitals are members of the European Reference Networks (ERNs), including the ERN-RND and the ERN-EYE which are dedicated to rare neurological diseases and rare eye diseases, because due to their rarity and complexity RDs often require extensive consultation, diagnostics and treatment, including surgery, often unavailable in Poland, there is a need to further develop international collaborative actions that enhance the accessibility of cross-border health care.
6. Since obtaining an RD diagnosis has a strong emotional impact on the entire family, tracking their mental health symptoms and supporting families with CRD should be a goal of health care and social services from the time of diagnosis through the entire trajectory of the disease. There is also a need to improve accessibility to patient advocacy and support groups.
7. As well as psychosocial support CRD families require financial support in access to genetic testing, modern medicines, specialised foods, rehabilitation and assistive equipment.
8. As well as the existing education programmes in university curricula in molecular biology, clinical genetics and diagnostics all healthcare professionals should also be trained in genetic testing and counselling to improve their understanding not only of the nature of RDs, but also their psycho-social impact on the family.
9. Healthcare professionals should recognise CRD parents as experts in their children’s disease and in the field of care.

### Supplementary Information

Supplementary Information 1.

Supplementary Information 2.

## Data Availability

The datasets generated during the study are available from the corresponding author on reasonable request.

## Abbreviations

CDR: Children with rare diseases
RD: Rare disease
