# Supplementary material for: Invisible patients in rare diseases: parental experiences with the healthcare and social services for children with rare diseases. A mixed method study
Source: Sci Rep. 2024 Jun 18;14:14016. doi: 10.1038/s41598-024-63962-4 (PMC11189503; doi:10.1038/s41598-024-63962-4)
Supplement: Supplementary file 2 — Supplementary Information 2. [file 41598_2024_63962_MOESM2_ESM.docx]

**Table S2. Supplementary material. List of rare disease****s**

| Name of disease | ORPHAcode | ICD-11 | Number of caregivers | Disease prevalence |
| --- | --- | --- | --- | --- |
| Achromatopsia | 49382 | 9B70 | 12 | 1 in 30 000-50 000 |
| Arnold-Chiari malformation type 1 | 268882 | LA07.4 | 5 | 1 in 2 000* |
| Coffin-Siris syndrome | 1465 | LD27·0Y | 6 | 1 in 1 000 000 |
| Cri du chat syndrome | 281 | LD44·51 | 6 | 1 in 15 000-45 000 |
| Cystic fibrosis | 586 | CA25 | 24 | 1 in 5 000-9 000 |
| Dravet syndrome | 33069 | 8A61·11 | 72 | 1 in 30 000 |
| Duchenne muscular dystrophy | 98896 | 8C70·1 | 38 | 1 in 3 500-9 300 |
| Ehlers-Danlos syndrome | 98249 | LD28·1 | 18 | 1 in 3 100-4 500* |
| Glycogen storage disease | 79201 | 5C51·3 | 12 | 1 in 20 000-43 000* |
| Haemophilia | 448 | 3B10·0 | 16 | 1 in 12 000 |
| Homocystinuria | 395 | E72·1 | 5 | 1 in 200 000-335 000 |
| Joubert syndrome | 475 | LD20·00 | 8 | 1 in 100 000 |
| Kabuki syndrome | 2322 | LD2F·1Y | 10 | 1 in 32 000 |
| Long-chain 3-hydroxyacyl-CoA dehydrogenase deficiency | 5 | 5C52·01 | 12 | 1 in 120 000-250 000 |
| Loeys-Dietz syndrome | 60030 | BD50·Z | 6 | 1 in 100 000* |
| Marfan syndrome | 558 | LD28·01 | 17 | 1 in 5 000 |
| Medium-chain acyl-CoA dehydrogenase deficiency | 42 | 5C52·01 | 9 | 1 in 14 600 |
| Mitochondriopatia | 102 | 8D87·0 | 5 | 1 in 20 000-50 000 |
| Mucopolysaccharidosis | 79213 | 5C56·3 | 6 | 1 in 100 000 |
| Noonan syndrome | 648 | LD2F·15 | 7 | 1 in 2 500 |
| Phenylketonuria, mild hyperphenylalaninemia | 79254 | 5C50·00, C50·0Y | 176 | 1 in 15 000 |
| Rett syndrome | 778 | LD90·4 | 7 | 1 in 11 100-100 000 |
| Rubinstein-Taybi syndrome | 783 | Q87·2 | 5 | 1 in 100 000-125 000 |
| Smith-Magenis syndrome | 819 | LD44·H1 | 9 | 1 in 15 000-25 000 |
| Sotos syndrome | 821 | LD2C | 18 | 1 in 14 000 |
| Turner syndrome | 881 | LD50·0 | 20 | 1 in 2 000 |
| Tuberous sclerosis | 805 | Q85·1 | 20 | 1 in 10 000 |
| Williams syndrome | 904 | Q93·8 | 33 | 1 in 7 500 |
| Wilson disease | 905 | 5C64·00 | 8 | 1 in 30 000-110 000 |
| Wolf-Hirschhorn syndrome | 280 | LD44·41 | 7 | 1 in 20 000-50 000 |
| 22q11.2 deletion syndrome | 567 | LD44·N0 | 44 | 1 in 4 500-10 000 |
| Other |  |  | 284 | -- |

Sources: [www.orpha.net](http://www.orpha.net), *<https://www.ncbi.nlm.nih.gov>, <https://www.ehlers-danlos.com>, [www.radiopaedia.org](http://www.radiopaedia.org).
